# Plasmalogens and Photooxidative Stress Signaling in Myxobacteria, and How it Unmasked CarF/TMEM189 as the Δ1′-Desaturase PEDS1 for Human Plasmalogen Biosynthesis

**DOI:** 10.3389/fcell.2022.884689

**Published:** 2022-05-11

**Authors:** S. Padmanabhan, Antonio J. Monera-Girona, Elena Pajares-Martínez, Eva Bastida-Martínez, Irene del Rey Navalón, Ricardo Pérez-Castaño, María Luisa Galbis-Martínez, Marta Fontes, Montserrat Elías-Arnanz

**Affiliations:** ^1^ Instituto de Química Física “Rocasolano”, Consejo Superior de Investigaciones Científicas, Madrid, Spain; ^2^ Departamento de Genética y Microbiología, Área de Genética (Unidad Asociada al IQFR-CSIC), Facultad de Biología, Universidad de Murcia, Murcia, Spain

**Keywords:** plasmalogens, *Myxococcus xanthus*, CarF, lipid signaling, singlet oxygen, photoregulation, TMEM189, PEDS1

## Abstract

Plasmalogens are glycerophospholipids with a hallmark *sn*-1 vinyl ether bond that endows them with unique physical-chemical properties. They have proposed biological roles in membrane organization, fluidity, signaling, and antioxidative functions, and abnormal plasmalogen levels correlate with various human pathologies, including cancer and Alzheimer’s disease. The presence of plasmalogens in animals and in anaerobic bacteria, but not in plants and fungi, is well-documented. However, their occurrence in the obligately aerobic myxobacteria, exceptional among aerobic bacteria, is often overlooked. Tellingly, discovery of the key desaturase indispensable for vinyl ether bond formation, and therefore fundamental in plasmalogen biogenesis, emerged from delving into how the soil myxobacterium *Myxococcus xanthus* responds to light. A recent pioneering study unmasked myxobacterial CarF and its human ortholog TMEM189 as the long-sought plasmanylethanolamine desaturase (PEDS1), thus opening a crucial door to study plasmalogen biogenesis, functions, and roles in disease. The findings demonstrated the broad evolutionary sweep of the enzyme and also firmly established a specific signaling role for plasmalogens in a photooxidative stress response. Here, we will recount our take on this fascinating story and its implications, and review the current state of knowledge on plasmalogens, their biosynthesis and functions in the aerobic myxobacteria.

## Introduction

**FIGURE 1:**
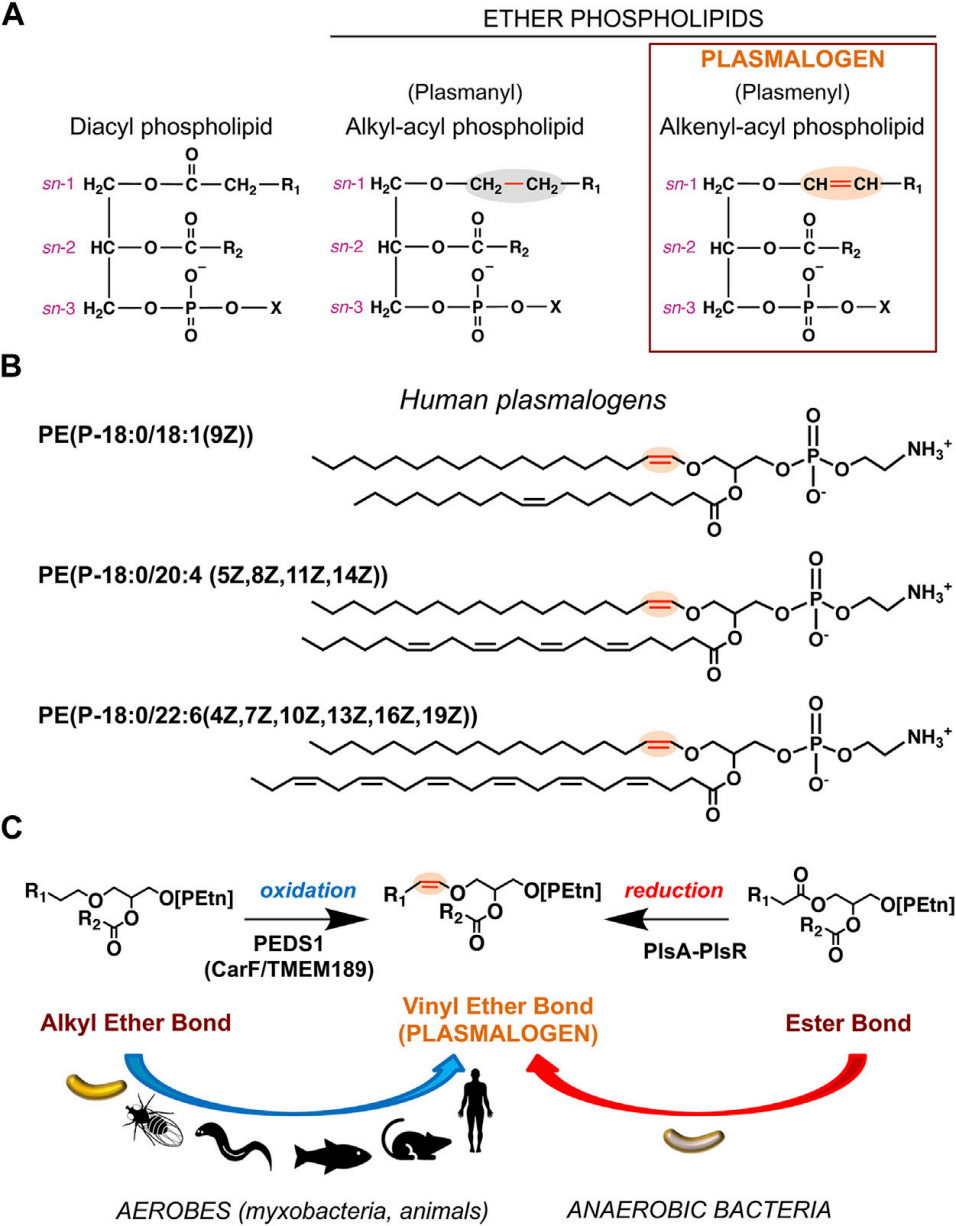
Chemical structures of plasmalogens and generation of the hallmark vinyl ether bond. **(A)** Chemical structures represented as Fischer projections of the ester-linked diacyl glycerophospholipids (left), alkyl ether lipids (middle), and vinyl ether lipids or plasmalogens (right). The vinyl ether and its precursor alkyl ether bond are highlighted in red and shaded orange and grey, respectively; *sn* positions are indicated; R_1_ and R_2_ represent hydrocarbon chains at the *sn*-1 and *sn*-2 positions, respectively; X = ethanolamine (Etn) or choline (Cho) moiety. **(B)** Representative human plasmalogens. PE (P-18:0/18:1 (9Z)) or 1-(1Z-octadecenyl)-2-(9Z-octadecenoyl)-sn-glycero-3-phosphoethanolamine; PE (P-18:0/20:4 (5Z,8Z,11Z,14Z)) or 1-(1Z-octadecenyl)-2-(5Z,8Z,11Z,14Z-eicosatetraenoyl)-sn-glycero-3-phosphoethanolamine (5Z,8Z,11Z,14Z-eicosatetraenoyl = arachidonyl); PE (P-18:0/22:6 (4Z,7Z,10Z,13Z,16Z,19Z)) or 1-(1Z-octadecenyl)-2-(4Z,7Z,10Z,13Z,16Z,19Z-docosahexaenoyl)-sn-glycero-3-phosphoethanolamine. LIPID MAPS Structure Database (https://www.lipidmaps.org/databases/lmsd) common and systematic names are used. **(C)** Vinyl ether bond formation in plasmalogens by the aerobic (oxidative) pathway in myxobacteria and animals and the anaerobic (reductive) route in anaerobic bacteria.

Membranes are vital barriers between a living cell and its environment, and between subcellular compartments, which ensure selective trafficking of substances, signal sensing and transduction, energy generation, and enable many other functions indispensable for cell viability (Harayama and Riezman, 2018). Their basic and predominant components are lipids of highly diverse chemical structures, composition, and distribution, depending on the organism, cell type or organelle. Lipids affect membrane properties, like rigidity and fluidity, and serve as platforms for other crucial biomolecules, like proteins and carbohydrates. Lipids are classified into eight categories based on the chemically functional backbone and further divided into classes and subclasses depending on their alkyl/acyl unit size, number and position(s) of double bond(s), hydroxylation, presence/type of polar headgroups, and presence of ester or ether bonds (Fahy et al., 2009). Plasmalogens, the theme of the current Research Topic, belong to a major membrane lipid category known as glycerophospholipids (GPs), defined by a glycerol backbone, one of whose hydroxyl groups is esterified to a phosphate (or phosphonate (Metcalf and van der Donk, 2009; Horsman and Zechel, 2017)) group at the *sn*-3 position (*sn*, stereospecific numbering). Typical GPs (or diacyl GPs) have both remaining hydroxyl groups linked through ester bonds to long-chain fatty acids, whereas ether GPs have an ether bonded alkyl chain at *sn*-1 and an ester bonded fatty acid at *sn*-2 (Figure 1A). Variants with a second ether-linked alkyl group at *sn*-2 reportedly occur in some bacteria (Caillon et al., 1983). The alkyl chains, especially in mammals, are 16 or 18 carbon atoms long, usually saturated (Figure 1B) or monounsaturated, while in bacteria they often have odd number of carbon atoms and can be saturated, unsaturated, and iso-branched (Kaneda, 1991; Rezanka and Sigler, 2009; Sohlenkamp and Geiger, 2016). Among ether lipids with an *sn*-2 fatty acyl group, plasmanyl GPs contain an *sn*-1 alkyl group (1-*O*-alkyl, linked by an ether bond), while plasmenyl GPs contain an *sn*-1 alkenyl group (1-*O*-alk-1′-enyl, with a double bond adjacent to the ether linkage or vinyl ether bond) and are known as plasmalogens (Figure 1A).

Plasmalogens were identified a century ago and so named as the source of “plasmal”, the aldehyde produced by acid treated cell plasma (Feulgen and Voit, 1924; Snyder, 1999). Their presence in animals and anaerobic bacteria is well established, as also their absence (save some stray reports) in plants and fungi (Braverman and Moser, 2012; Goldfine, 2017; Zhou et al., 2020; Vítová et al., 2021). Obligate or facultative aerobic bacteria are often described as lacking plasmalogens but this ignores a striking exception, the obligately aerobic myxobacteria, where the presence of plasmalogens was first reported 50 years ago (Kleinig, 1972) and confirmed in subsequent studies (Stein and Budzikiewicz, 1987; Curtis et al., 2006; Ring et al., 2006; Lorenzen et al., 2014a; Lorenzen et al., 2014b; Gallego-García et al., 2019). When present, plasmalogens often exceed other ether lipids in abundance and can constitute as much as a fifth of the mammalian phospholipidome (Braverman and Moser, 2012). Their composition varies with organisms, organelles, and cell types. Enriched in the brain, heart, kidney, lung, skeletal muscle, and neutrophils but low in the liver, plasmalogens occur in almost all subcellular membranes except in peroxisomes, the site of the early steps in their biosynthesis (Braverman and Moser, 2012). Their levels fluctuate with age, growth, and environmental conditions and reflect a balance between biosynthesis and degradation. In mammals, the first step in their biosynthesis, the generation of a fatty alcohol by FAR1, a peroxisome-associated fatty alcohol reductase that preferentially reduces C16 and C18 fatty acyl-CoAs (see below), is proposed to be rate-limiting. Plasmalogen levels appear to modulate feedback regulation as well as degradation of FAR1, but the underlying molecular mechanisms for sensing, signaling and spatiotemporal regulation remain undetermined (Honsho and Fujiki, 2017). Moreover, crosstalk and co-regulation of ether lipids and plasmalogens with other lipid classes form an important part of the complex interconnections in membrane lipid metabolism and homeostasis networks (Jiménez-Rojo and Riezman, 2019). In bacteria, lipids and their compositions also vary with species, growth and environmental conditions (Sohlenkamp and Geiger, 2016). For example, in the aerobic plasmalogen-producing soil myxobacterium *Myxococcus xanthus*, large shifts in lipid composition accompany the starvation-induced development of vegetatively growing cells into multicellular spore-filled fruiting bodies (Lorenzen et al., 2014b; Ahrendt et al., 2015).

Abnormalities and deficiencies in ether lipid/plasmalogen levels have been linked to various human pathologies ranging from rare genetic disorders (Rhizomelic chondrodysplasia punctata, Zellweger syndrome) to cancer, neurodegenerative diseases like Alzheimer’s disease, and metabolic disorders (Braverman and Moser, 2012; Dean and Lodhi, 2018; Paul et al., 2019). Recently, plasmalogens were implicated in cell fitness under hypoxia (low oxygen) conditions (Jain et al., 2020) and in ferroptosis (Zou et al., 2020; Cui et al., 2021). The special physicochemical attributes of the plasmalogen vinyl ether bond can affect membrane fluidity, signaling and function (Braverman and Moser, 2012; Jiménez-Rojo and Riezman, 2019), and its susceptibility to cleavage by reactive oxygen species (ROS) such as singlet oxygen (^1^O_2_) yields products that may act as second messengers (Morand et al., 1988; Zoeller et al., 1988; Stadelmann-Ingrand et al., 2001; Jenkins et al., 2018; Gallego-García et al., 2019). Membrane microdomain platforms called lipid rafts, proposed to recruit and activate signaling proteins, are enriched in plasmalogens (Pike et al., 2002; Braverman and Moser, 2012; Dean and Lodhi, 2018). Hence, plasmalogens have been associated with antioxidative and signaling mechanisms. Yet, despite their abundance, links to disease and distinctive properties, studies on plasmalogens, although rising in recent years, remain modest when compared to other lipid classes, and their exact biological functions and the molecular basis of their actions continue to be enigmatic.

Multistep pathways have been charted for the aerobic plasmalogen biosynthesis in mammals and for a different oxygen-independent route in anaerobic bacteria, with enzymes for most of the proposed steps identified (Braverman and Moser, 2012; Goldfine, 2017; Vítová et al., 2021). Even so, the key enzymes that generate the characteristic plasmalogen vinyl ether bond in the final steps of both pathways remained unknown and were discovered only very recently (Figure 1C). A year ago, one study identified a two-gene bacterial operon encoding a multidomain complex for the anaerobic reduction-dehydration that converts the *sn*-1 ester bond of a diacyl lipid into a vinyl ether one (Jackson et al., 2021). It also reported that diverse facultative anaerobic bacteria, including many human gut microbes, can and do make plasmalogens, not just obligate anaerobes as suggested earlier (Goldfine, 2010). Two years ago, work on photooxidative stress signaling in the obligately aerobic *M. xanthus* led to the discovery that the oxygen-dependent desaturase for plasmalogen biosynthesis is a membrane protein called CarF, and that this protein is functionally conserved in sequence homologues denoted TMEM189 in human, mouse, zebrafish, fruit fly or nematodes (Gallego-García et al., 2019). The unmasking of this mammalian enzyme, now named PEDS1 and sought for five decades, was reinforced further in two subsequent reports (Werner et al., 2020; Wainberg et al., 2021). Besides revealing the identity of a key enzyme for aerobic plasmalogen biogenesis and its remarkable evolutionary conservation from bacteria to humans, the study in *M. xanthus* unearthed a specific signaling role for plasmalogens in directing a response to ^1^O_2_ and photooxidative stress. We discuss below these aspects of aerobic plasmalogen biosynthesis and signaling roles in myxobacteria.

## *Myxococcus xanthus* and Myxobacteria- an Overview

*M. xanthus* is a Gram-negative soil bacterium and the best studied member of myxobacteria. These constitute the order Myxococcales, currently in the Deltaproteobacteria class of the phylum Proteobacteria, but recently proposed for reclassification as a new phylum named Myxococcota, with the order comprising three suborders (Cystobacterineae, Sorangiineae, Nannocystineae) and 10 families (Mohr, 2018; Vos, 2021; Pérez-Castaño et al., 2022). Widely distributed, most myxobacteria are soil-dwelling, though examples in aquatic environments are also known, and all are obligate aerobes except for the facultative anaerobic genus *Anaeromyxobacter*. Their GC-rich (66–75%) genomes are among the largest in bacteria (∼9–16 Mb; 9.14 Mb and around 7,500 genes in *M. xanthus*) but reduced with extensive gene loss in *Anaeromyxobacter* (∼4.4 Mb) and *Vulgatibacter incomptus* (∼5 Mb). They are an important source, albeit not fully tapped, of many unique bioactive compounds and secondary metabolites that are often drug leads for pharmaceutical applications (Bader et al., 2020). Myxobacteria are striking in their complex lifestyles, with some traits usually attributed to eukaryotes, like multicellular development, social cooperative behavior, kin recognition, predation, motility and, pertinent to this review, biosynthesis of specialized lipids and steroids (Muñoz-Dorado et al., 2016; Cao and Wall, 2019; Gallego-García et al., 2019; Hoshino and Gaucher, 2021). These aspects have been investigated in *Myxococcus xanthus* that, as a model bacterial system to study light sensing and response, has uncovered new photosensory transduction and gene regulation paradigms (Elías-Arnanz et al., 2011; Padmanabhan et al., 2021), including the aforementioned conserved desaturase for plasmalogen biosynthesis (Gallego-García et al., 2019).

## Mammalian Aerobic Plasmalogen Biosynthesis

**FIGURE 2:**
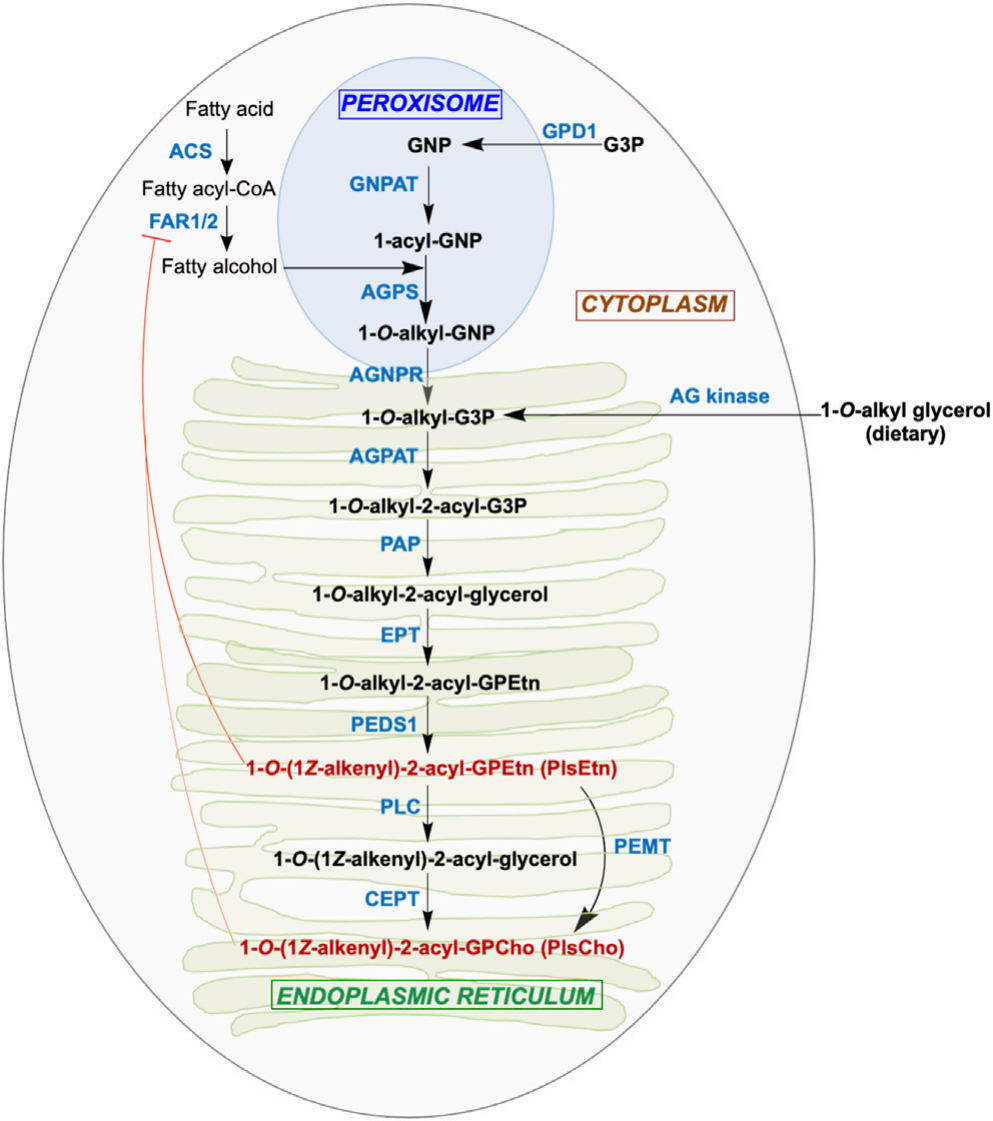
Aerobic plasmalogen biosynthesis pathway in mammals. Synthesis of the major ethanolamine form of the plasmalogen (PlsEtn) begins in the peroxisomes and ends in the ER, followed by transport to post-Golgi compartments. The substrate and product (black boldface), abbreviated enzyme names (blue boldface; see text for full names and Enzyme Comission numbers), the organelle where each step takes place, and feedback regulation of FAR1 by plasmalogens (red blunt ended arrow) are shown.

An obligate aerobe with the same enzyme PEDS1 for a key late step in plasmalogen biogenesis as in animals, *M. xanthus* may share other parallels with the mammalian pathway. The latter (Figure 2), which has been reviewed elsewhere (Nagan and Zoeller, 2001; Braverman and Moser, 2012; Dean and Lodhi, 2018; Zhou et al., 2020; Vítová et al., 2021), begins in the peroxisome and ends in the endoplasmic reticulum (ER). The earliest steps in the peroxisomal lumen use as substrates: 1) imported dihydroxyacetone phosphate (DHAP) or glycerone phosphate (GNP), derived from glycerol 3-phosphate (G3P), possibly catalyzed by glycerol 3-phosphate dehydrogenase 1 (GPD1; Enzyme Comission number EC 1.1.1.8); 2) long-chain (C16, C18) fatty alcohols generated by the action of fatty acyl-CoA reductase (FAR1 or the less broadly distributed FAR2; EC 1.2.1.84) on fatty acyl-CoA, produced from fatty acid by acyl-CoA synthase (ACS; EC 6.2.1.3). FAR1 is tail anchored to the peroxisome membrane cytosolic face and is also found in lipid droplets (Exner et al., 2019). Glycerone phosphate *O*-acyltransferase (GNPAT; EC 2.3.1.42) converts GNP to 1-acyl-GNP, whose acyl chain is then replaced by a fatty alcohol to yield 1-*O*-alkyl-GNP (AGP) in a reaction catalysed by alkylglycerone-phosphate synthase (AGPS; EC 2.5.1.26). GNPAT and AGPS function as a complex to optimize substrate channeling and catalytic efficiency. In the next step, the acylglycerone-phosphate reductase (AGNPR or PexRAP, for peroxisomal reductase activating PPARγ; EC 1.1.1.101), localized in the ER and peroxisomal membranes (Lodhi et al., 2012; Honsho et al., 2020), reduces 1-*O*-alkyl-GNP to 1-*O*-alkyl-G3P, which can also be supplied by phosphorylation of dietary 1-*O*-alkylglycerol (AG) by alkylglycerol kinase (AG kinase; EC 2.7.1.93) (Snyder, 1992). Exchange of 1-*O*-alkyl-G3P and other lipids between peroxisomes and the ER is mediated by peroxisome-ER tethering, which relies on the interactions of the peroxisomal membrane protein ACBD5 (acyl-CoA binding domain containing 5) with cytosolic regions of the ER-resident VAMP-associated proteins VAPA and VAPB (Hua et al., 2017).

The remaining steps in the ER to produce plasmalogens mirror those for ester-linked diacyl GP biosynthesis from diacyl-G3P. Acylation at the *sn*-2 position of 1-*O*-alkyl-G3P by an alkylglycerolphosphate 2-*O*-acyltransferase (AGPAT; EC 2.3.1.-) yields 1-*O*-alkyl-2-acyl-G3P, whose phosphate group removal by phosphatidate phosphatase (PAP; EC 3.1.3.4) produces 1-*O*-alkyl-2-acyl-glycerol. To this, ethanolamine (Etn) phosphotransferase (EPT; EC 2.7.8.1) adds a phospho-Etn group from cytidine diphosphate ethanolamine to produce 1-*O*-alkyl-2-acyl-GPEtn. This alkyl ether (plasmanyl) lipid is the substrate for the oxygen and cytochrome *b5*-dependent TMEM189 or plasmanylethanolamine desaturase (PEDS1; EC 1.14.19.77), which generates the vinyl ether bond in PlsEtn, the Etn plasmalogen. Conversion of PlsEtn to the choline (Cho) form, PlsCho, is achieved either by phosphatidylethanolamine N-methyltransferase (PEMT; EC 2.1.1.17) or by removal of phospho-Etn by phospholipase C (PLC; EC 3.1.4.3) and phospho-Cho addition by choline/ethanolamine phosphotransferase 1 (CEPT; EC 2.7.8.2). In most mammalian tissues, PlsEtn generally predominates and is longer-lived than PlsCho (Braverman and Moser, 2012; Messias et al., 2018). Although the above enzymatic steps were established early on, some of the actual enzymes involved were unknown and described as “orphan” (Watschinger and Werner, 2013). The identities of some of these, like TMEM189, or the exact roles and cellular locations of others, like EPT/CEPT, were established just recently (Horibata et al., 2020), which presages that new players with possibly new roles may emerge in future studies.

Regulation of plasmalogen biosynthesis and metabolism would be expected at several levels, given the multiple enzymes involved, the need for coordinated transport of precursors and products to target organelles and membranes, and links to degradation and turnover. Feedback regulation and degradation of FAR1 (hence, fatty alcohol substrate availability) by cellular plasmalogen levels, and crosstalk and co-regulation of ether lipids/plasmalogens and some other lipid classes, have been mentioned (Honsho and Fujiki, 2017; Jiménez-Rojo and Riezman, 2019). But in contrast to many other lipid classes, transcriptional and post-translational regulatory mechanisms in plasmalogen biosynthesis remain largely unknown. The plasmalogen vinyl ether bond can be cleaved by plasmalogenase activity of cytochrome *c* in the presence of cardiolipin, O_2_ and H_2_O_2_, or oxidized cardiolipin and O_2_, to produce 2-acyl-lysophospholipids and α-hydroxy fatty aldehydes; or by acidic HOCl or HOBr generated by leukocyte myeloperoxidase to yield α-haloaldehydes (Jenkins et al., 2018; Ebenezer et al., 2020). The vinyl ether bond in lysoplasmalogen, lacking the *sn*-2 acyl group, is reportedly targeted by lysoplasmalogenase (TMEM86B), which is localized at the cytoplasmic membrane (Wu et al., 2011). On the other hand, ER membrane-localized alkylglycerol monooxygenase (AGMO or TMEM195) cleaves the alkyl (not vinyl) ether bond in alkylglycerols or lyso-alkylGPs to generate a fatty aldehyde and the corresponding glycerol derivative (Watschinger et al., 2010). Although there is evidence that these degradation products may act as signaling molecules or second messengers, many questions remain, including their possible links to plasmalogen biosynthesis (Dorninger et al., 2020; Ebenezer et al., 2020). In short, regulation of plasmalogen biosynthesis may occur at multiple levels but most of the mechanisms involved remain to be elucidated.

## Aerobic Plasmalogen Biosynthesis in Myxobacteria

**FIGURE 3:**
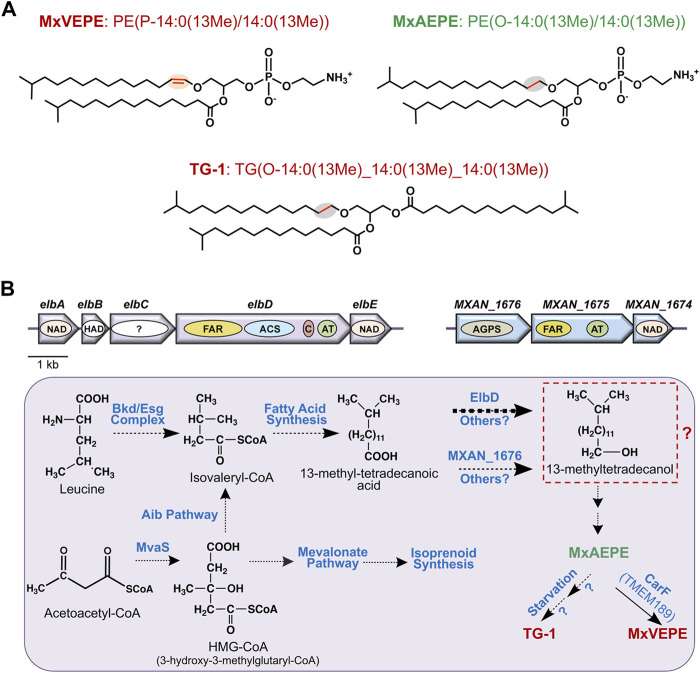
*M. xanthus* alkyl ether lipids, plasmalogens and their biosynthesis. **(A)** *M. xanthus* plasmalogen MxVEPE: 1-(13-methyl-1Z-tetradecenyl)-2-(13-methyl-tetradecanonyl)-sn-glycero-3-phosphoethanolamine; its alkyl ether (plasmanyl) precursor MxAEPE: 1-(13-methyl-tetradecanyl)-2-(13-methyl-tetradecanoyl)-sn-glycero-3-phosphoethanolamine; and the alkyldiacylglycerol ether lipid TG-1: rac1,2-di-(13-methyltetradecanoyl)-3-(13-methyltetradecyl)-glycerol. LIPID MAPS Structure Database (https://www.lipidmaps.org/databases/lmsd) systematic and common names are used. **(B)** Plasmalogen biosynthesis pathway in *M. xanthus* highlighting known and proposed steps. Top: The *elbA-elbE* and the *MXAN_1676-MXAN_1674* operons. Arrows indicate genes and the ovals inside depict proteins or putative domains. AGPS, FAR, and ACS are as in Figure 2, AT is acyltransferase (AGPAT/GNPAT-like); C is acyl-carrier protein or ACP; NAD is an NAD-dependent epimerase/dehydratase family protein; “?” is a protein domain of unknown function. Bottom: Proposed and known steps in *M. xanthus* plasmalogen biosynthesis. Broken arrows indicate multiple steps involved, and the thick arrow for ElbD indicates this pathway is predominant. Steps from the iso-branched fatty acid to the alkyl ether lipid MxAEPE are unknown and may involve hypothetical conversion to a fatty alcohol (red dashed box) as in the mammalian aerobic plasmalogen biosynthesis pathway.

Compared to mammals, far less is known about ether lipid or plasmalogen biosynthesis in aerobic myxobacteria. In the best-studied *M. xanthus*, a complete data set for molecular lipid species was reported less than a decade ago (Lorenzen et al., 2014a; Lorenzen et al., 2014b) and culminated preceding studies (Curtis et al., 2006; Ring et al., 2006; Hoiczyk et al., 2009). The predominance of iso-branched fatty acids (FAs) in *M. xanthus* and some other myxobacteria was described 50 years ago (Ware and Dworkin, 1973), as also the presence of alkyl-acyl and alk-1′-enyl-acyl phosphoglycerides (Kleinig, 1972), with the latter shown to correspond to plasmalogens over a decade later (Stein and Budzikiewicz, 1987). The principal plasmalogen, denoted MxVEPE, and the corresponding alkyl ether plasmanyl form (MxAEPE) are both Etn GPs (*M. xanthus* is devoid of Cho GPs) with iso-branched C15 (i15:0) chains at both the *sn*-1 and *sn*-2 positions, although low levels of i17:0 can be found at *sn*-2 (Figure 3A). Under both vegetative and starvation conditions, MxVEPE far exceeds MxAEPE (Lorenzen et al., 2014a; Lorenzen et al., 2014b). Moreover, during starvation, which induces fruiting body formation, neutral ether lipids like 1-*O*-alkyl-*sn*-glycerol, 1-*O*-alkyl-2-acyl-*sn*-glycerol and, primarily, 1-*O*-alkyl-2,3-diacyl-*sn*-glycerol (TG-1; Figure 3A), accumulate in lipid bodies (Ring et al., 2006; Hoiczyk et al., 2009). TG-1, rare in nature like other diacylglycerol ether lipids, is a reported signal for *M. xanthus* fruiting body development (Bhat et al., 2014a).

Even though the identity of the Δ1′-desaturase required for aerobic plasmalogen synthesis was first unmasked in *M. xanthus* (Gallego-García et al., 2019), most other steps and relevant enzymes in the pathway remain uncharacterized. A feature of the mammalian pathway is the distribution of defined steps among organelles. This is unlikely in a prokaryote like *M. xanthus*, but there is increasing evidence that microcompartments or organelles in bacteria, including myxobacteria, can sequester and control specific metabolic pathways (Kerfeld et al., 2018; Greening and Lithgow, 2020). The formation of iso-branched FAs required early in *M. xanthus* plasmalogen biosynthesis (Figure 3B), which has been studied in some detail, occurs through two-carbon extensions of the starting unit isovaleryl-CoA to full length via the fatty acid synthase cycle and several proposed enzymes (Bhat et al., 2014b). Two distinct pathways generate isovaleryl-CoA, whose levels fall drastically when both routes are disrupted. One is the standard pathway for leucine degradation (also for valine or isoleucine) by the multisubunit branched-chain α-keto acid dehydrogenase (Bkd; or Esg, from E-signal) complex (Toal et al., 1995). A two-gene operon encodes the E1α and E1β α-ketoacid dehydrogenase subunits, while two proximal genes at another genomic locus encode the E2 dihydrolipoamide acetyl transacylase and E3 dihydrolipoamide dehydrogenase subunits. Transcription of these genes, which would be important in the regulation of branched-chain fatty acid synthesis, and thereby of ether lipids, depends on the growth medium (Bartholomeusz et al., 1998), but the molecular mechanisms involved remain unresolved. The alternative isovaleryl-CoA biosynthesis (Aib) pathway is an offshoot, possibly unique to myxobacteria, of the well-known mevalonate pathway for isoprenoid biosynthesis. It involves a gene for a 3-hydroxy-3-methylglutaryl coenzyme A dehydratase (LiuC) and a five-gene Aib operon. The latter operon encodes a transcriptional regulator (AibR), the 3-hydroxy-3-methylglutaryl coenzyme A synthase (MvaS), two subunits of a novel type of decarboxylase (AibA and AibB), and a medium-chain reductase-dehydrogenase (AibC). AibR, when bound to isovaleryl-CoA, downregulates the Aib operon and a gene encoding a putative acetyl-CoA acetyltransferase that likely catalyzes formation of the MvaS substrate acetoacetyl-CoA (Mahmud et al., 2002; Bode et al., 2006; Bode et al., 2009; Li et al., 2013; Bock et al., 2016; Bock et al., 2017a; Bock et al., 2017b). Interestingly, Aib pathway genes are upregulated upon starvation (when Bkd pathway genes are downregulated), and also in *bkd* mutants (Bode et al., 2009; Bhat et al., 2014b; Muñoz-Dorado et al., 2019), presumably to ensure supply of isovaleryl-CoA for iso-branched fatty acid synthesis-homeostasis under different conditions.

The pathway from iso-branched fatty acids to plasmalogens and the enzymes involved are unknown, except for the final steps, which emerged from two key recent studies. The first (Lorenzen et al., 2014a) described a multidomain protein, ElbD, encoded in a five-gene ether lipid biosynthesis *elb* operon (Figure 3B). Its study stemmed from examining the *M. xanthus* genome for genes encoding enzymes akin to ones known in plasmalogen biosynthesis. Thus, ElbD was annotated as a putative long-chain-fatty-acid CoA ligase with an N-terminal domain similar to FAR1, which generates fatty alcohol from fatty acid early in the mammalian pathway, and domains for ACS, which produces the FAR1 substrate, for acyl–carrier protein (ACP) and for an AGPAT-like acyltransferase (Figures 2, 3B). The *elbD* gene is flanked by *elbC*, which encodes a protein of unknown function, and by *elbE*, which encodes an NAD-dependent epimerase/dehydratase family protein, as does *elbA*. The fifth gene in the operon, *elbB,* encodes a protein of the haloacid dehalogenase (HAD)-like phosphohydrolase superfamily, whose members have diverse functions. The *M. xanthus* genome also revealed a three-gene operon (Figure 3B) comprising: 1) *MXAN_1676*, whose product is ∼42% identical (92% coverage) in sequence to human AGPS, which acts in the second peroxisomal step of ether lipid biosynthesis (Figure 2); 2) *MXAN_1,675*, encoding a protein with an N-terminal FAR1-like domain and a C-terminal GNPAT-like acyltransferase domain; 3) *MXAN_1,674,* encoding a putative NAD-dependent epimerase/dehydratase family protein, like ElbA and ElbE. The similarities of MXAN_1,675 and MXAN_1676 to eukaryotic enzymes and their possible implication in *M. xanthus* ether lipid biosynthesis had been noted and suggested, but not established, in an earlier study (Curtis et al., 2006). Disrupting *MXAN_1676*, however, caused only a minor reduction in ether lipids, suggesting an alternative pathway that was shown to depend on ElbD (Lorenzen et al., 2014a).

Insertion mutagenesis of *elbD* was found to strongly reduce vinyl and alkyl ether lipid content under both vegetative and starvation conditions and was rescued by complementation with *elbD* (Lorenzen et al., 2014a). Since insertions in *elbA* had no effects, ElbA was considered to be uninvolved in ether lipid synthesis. Insertions in *elbB* reduced vinyl and alkyl ether lipid levels, though not as much as in *elbD* mutants, and was partially restored by expression of *elbD*. This was attributed to polar effects of the *elbB* disruption. Finally, inactivating *elbE* decreased ether lipid formation and was not complemented by an extra copy of *elbD*. The study thus concluded that ElbD, and possibly ElbE, were important in *M. xanthus* ether lipid and plasmalogen biosynthesis. ElbD and a truncated form lacking the N-terminal putative FAR1-like domain could be purified as soluble proteins, which suggests ElbD is cytosolic. Analysis *in vitro* confirmed the ACP and ACS activities for these two ElbD domains, but the FAR1 and AGPAT domains could not be evaluated since no acyltransferase protein activity was detected with isolated ElbD, and fatty alcohols linked to FAR activity were undetected (Lorenzen et al., 2014a). Because an i15:0 aldehyde could be identified in wild-type strains but not in *elbD* mutants, it was proposed that the ElbD acyl-CoA reductase activity was to reduce CoA-bound fatty acid to the aldehyde, and that this was somehow involved in vinyl ether bond formation. Since only alkenyl and not alkyl Etn GPs were detectable in *M. xanthus* under vegetative and developmental conditions, a “reductive” ether lipid formation pathway was considered, with conversion of MxVEPE to MxAEPE to TG-1 (Ring et al., 2006; Lorenzen et al., 2014a). Such a route, which drew upon clues from the anaerobic pathway, would be unprecedented and is now implausible in light of the discovery that CarF drives aerobic oxidation of MxAEPE to MxVEPE (Figure 1C).

Lipid profiles along the *M. xanthus* life cycle have indicated that alkyl ether lipids accumulate only during development, when TG-1 reaches high levels while MxVEPE decreases gradually from ∼10% relative abundance in vegetative cells to ∼4% in mature fruiting bodies (Lorenzen et al., 2014a; Lorenzen et al., 2014b; Ahrendt et al., 2015). By comparison, the most abundant diacyl Etn GP decreases far more sharply, to 5% of the vegetative levels. Ether lipid metabolism has thus been inferred to exhibit differences in regulation and biosynthesis from the other lipids. Conclusive data are, however, needed regarding if and how the *elb* and *MXAN_1676* operons are regulated and what layers of regulation beyond transcription come into play. Identification of ElbD as a key player in *M. xanthus* alkyl ether lipid and plasmalogen biosynthesis provided a crucial launch point for further investigations, but many questions persist on the exact functions and biochemical mechanisms of ElbD and on the other players mentioned above. The recent finding that none of these generate the vinyl ether bond and that this task falls on CarF was therefore an important breakthrough.

## Photosensory Signaling in *M. xanthus* Carotenoid Synthesis, CarF and its Early Characterization

**FIGURE 4:**
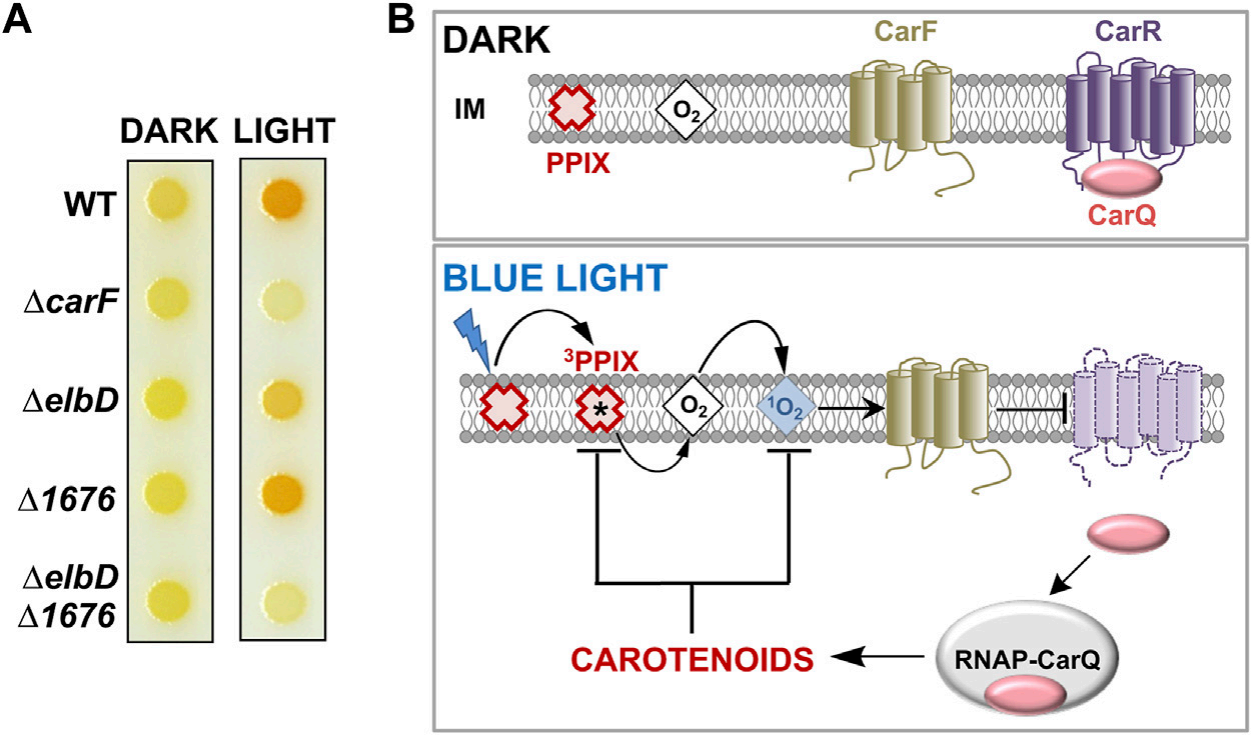
Light response in *M. xanthus*. **(A)** *M. xanthus* colony color in the dark and in the light in the wild-type strain (WT) and with various indicated deletions (adapted from (Gallego-García et al., 2019)). **(B)** Simplified model for the blue light-PPIX-^1^O_2_ signal transduction pathway in *M. xanthus*. In the dark, an inner membrane (IM) anti-σ factor (CarR) with a six transmembrane-helix topology sequesters its cognate *σ* factor (CarQ). Blue light excites protoporphyrin IX (PPIX), the iron-free heme precursor and photosensitizer, to the high-energy ^3^PPIX state, from which energy transfer to O_2_ generates highly reactive ^1^O_2_, which requires CarF to inactivate CarR by an unknown mechanism. This leads to the release of CarQ, which associates with RNA polymerase (RNAP) to activate specific genes enabling the synthesis of carotenoids to quench reactive ^1^O_2_ and ^3^PPIX and limit photooxidative damage (black arrows: activation; blunt-ended arrows: inactivation).

The surprising road to unmasking CarF in *M. xanthus* and its homologue TMEM189 in metazoa as the conserved Δ1′-desaturase for plasmalogen formation was paved in studies that examined how *M. xanthus* responds to light by synthesizing carotenoids, which quench ^1^O_2_ produced upon illumination and thereby protect cells against photooxidative damage (Padmanabhan et al., 2021). Carotenoids are lipophilic isoprenoid pigments with extended, all-trans, conjugated polyene chains (usually C40, sometimes C50, C45 and C30 terpenes) with acyclic, monocyclic, or bicyclic ends. Their synthesis in *M. xanthus* is visibly reflected by cells that are yellow in the dark (due to noncarotenoid pigments) turning to an orange-red color upon exposure to light (Figure 4A).

Two modes of light sensing and signaling operate in *M. xanthus* to trigger a transcriptional response leading to carotenoid biosynthesis (Padmanabhan et al., 2021; Pérez-Castaño et al., 2022). In one, the photoreceptor protein CarH, with coenzyme B_12_ as its chromophore, directly senses UV, blue or green light and turns on transcription of genes encoding enzymes required for carotenogenesis (Ortiz-Guerrero et al., 2011; Jost et al., 2015; Padmanabhan et al., 2017; Padmanabhan et al., 2019; Padmanabhan et al., 2022). In the other mechanism (Figure 4B), blue light excites the iron-free heme precursor protoporphyrin IX or PPIX, a hydrophobic cyclic tetrapyrrole and photosensitizer that accumulates in the *M. xanthus* cell membrane especially during stationary phase (Burchard and Dworkin, 1966; Galbis-Martínez et al., 2012). PPIX photoexcitation generates the high-energy ^3^PPIX triplet state, which can transfer energy to molecular oxygen yielding the extremely reactive and cytotoxic ^1^O_2_, a relatively long-lived and diffusible ROS in membrane environments (Ziegelhoffer and Donohue, 2009; Glaeser et al., 2011). This photoinduced ^1^O_2_ inactivates CarR (with six transmembrane helices) by an unknown mechanism to liberate CarQ, which is sequestered specifically at the membrane via its physical interaction with CarR. Free CarQ, in complex with RNA polymerase, activates transcription of genes for carotenoid synthesis. CarR belongs to a class of bacterial regulators known as anti-σ factors, which negatively control cognate factors known as extracytoplasmic function or ECF *σ* factors. Generally, specific signals inactivate a given anti-σ to liberate its cognate ECF *σ*, which associates with RNA polymerase to recognize defined promoters and initiate transcription of the corresponding genes (de Dios et al., 2021). The quest to elucidate how light and ^1^O_2_ cause inactivation of CarR led to the discovery of CarF.

The *carF* gene was identified two decades ago (Fontes et al., 2003) by genetic analysis of *M. xanthus* mutants devoid of light-induced carotenogenesis (hence the *car* nomenclature). Strains with *carF* disrupted or deleted (Δ*carF*) failed to produce carotenoids and therefore did not acquire the characteristic orange-red color in the light (Figure 4A). Moderate and steady levels of expression of *carF* over a wide range of conditions (dark or light, vegetative or stationary phase) were observed, and epistatic analysis established that CarF acted earlier than CarR in the signaling cascade (Fontes et al., 2003). The 281-amino acid CarF sequence was found to lack significant similarity to any bacterial or archaeal protein. But it strongly resembled (46% sequence identity, 59% similarity over a 237-residue stretch) a human protein of unknown function at the time that was named Kua (Thomson et al., 2000), but is now better known as TMEM189. CarF/Kua homologues were found in mouse (*Mus musculus*), zebrafish (*Danio rerio*), fruit fly (*Drosophila melanogaster*), worm (*Caenorhabditis elegans*), protozoa (*Leishmania*), and plants (*Arabidopsis thaliana*). The human gene for Kua/TMEM189 lies adjacent to *UEV1*, which encodes an inactive variant of E2 ubiquitin-conjugating enzymes. *Kua* and *UEV1* were found to be expressed as separate transcripts for the individual Kua and UEV1 proteins, but also as a rare hybrid transcript for a two-domain Kua-UEV1 fusion protein. The fusion product was also observed in rhesus monkey and chimpanzee but not in mice or non-mammals like *C. elegans* or *D. melanogaster* (Thomson et al., 2000; Duex et al., 2010). The Kua and CarF protein sequences suggested that they would be membrane proteins, with several histidines (see below) distributed in a pattern reminiscent of diiron histidine-rich motifs found on the luminal side of membrane fatty acid desaturases and hydroxylases (Shanklin et al., 2009). Interest in human Kua was, however, more centered on this rare inactive UEV fusion. Indeed, Kua homologues figured only as a UEV1 localization domain in a protein family PF10520 described as Kua-UEV1_localn; and their true function remained elusive even after a *Tmem189* knockout mouse was generated in a large-scale international mouse phenotyping consortium and several associated phenotypes, such as decreased body mass, and eye, bone and blood abnormalities were described (White et al., 2013).

What role CarF played in light-regulated carotenogenesis also remained enigmatic. It was demonstrated experimentally that CarF is a four-transmembrane helix topology protein that self-interacted and was essential in mediating the transmission of the ^1^O_2_ signal in the trajectory from blue light to CarR inactivation (Figure 4B) (Galbis-Martínez et al., 2008; Galbis-Martínez et al., 2012). Nevertheless, the molecular interplay between CarF and ^1^O_2_, whether it was direct or it involved one or more intermediary molecules still remained unanswered. How this was addressed and uncovered CarF function is described next.

## Unmasking CarF/TMEM189 as the Conserved Δ1′-Desaturase for Vinyl Ether Bond Formation in Plasmalogen Biosynthesis

**FIGURE 5:**
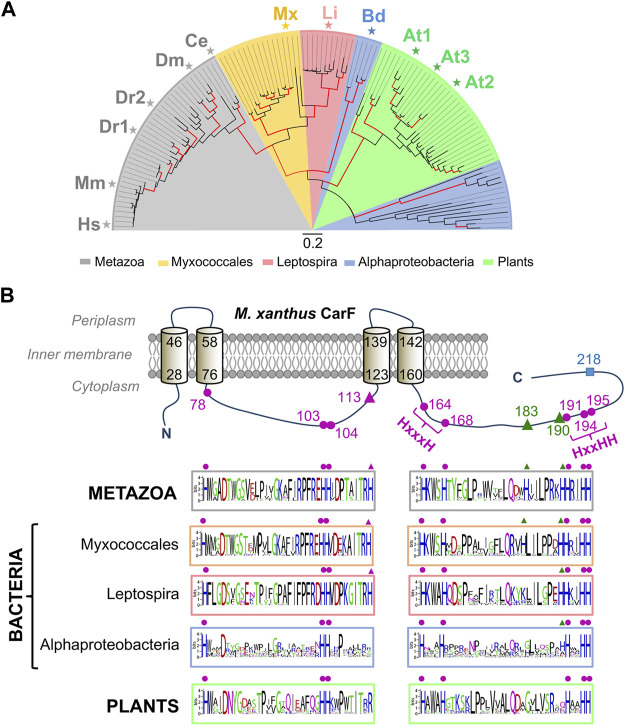
Phylogenetic distribution and conserved histidines of CarF homologues. **(A)** Phylogenetic tree for CarF homologues in metazoa, bacteria and plants (maximum-likelihood with red branches for ≥ 75% confidence from 200 bootstrap replicates; scale bar, number of substitutions per residue; key below for colored sectors; adapted from (Padmanabhan et al., 2021). Homologues that were tested for CarF function in *M. xanthus* are indicated: Hs, *Homo sapiens*; Mm, *M. musculus*; Dr, *D. rerio* (two homologues); Dm, *D. melanogaster*; Ce, *C. elegans*; Mx, *M. xanthus*; Li, *Leptospira interrogans*; Bd, *Bradyrhizobium diazoefficiens*; At, *A. thaliana* (three homologues). **(B)** Top: *M. xanthus* CarF cartoon representation with its experimentally established four transmembrane-helix topology (helix delimiting residues numbered) and twelve histidines (all cytoplasmic and numbered). CarF histidines conserved in all homologues, whose mutation abolishes function, are depicted by the magenta dots; the one histidine in CarF present in homologues from metazoa, myxobacteria and *Leptospira* and also essential for function is depicted by the magenta triangle; the two histidines conserved only in some homologues (primarily, from metazoa and myxobacteria), whose mutation has no effect, are depicted as green triangles; the non-conserved and non-essential CarF histidine (H218) is depicted as a cyan square. Bottom: sequence logos for segments containing the conserved and semi-conserved histidines in CarF homologues from metazoa, bacteria and plants (histidines are aligned with the equivalent ones in the CarF cartoon and marked using the same symbols; adapted from (Gallego-García et al., 2019).

An extensive search for homologues across a wide range of organisms revealed that CarF was confined to very few bacteria, mainly myxobacteria and a few *Leptospira* and Alphaproteobacteria, and that it was absent in archaea (Gallego-García et al., 2019). By contrast, CarF homologues were very prevalent among eukaryotes, invertebrate or vertebrate animal species, and also in plants, but very rare in fungi. Phylogenetic analysis based on more than a hundred homologues suggested that myxobacterial and *Leptospira* CarF homologues are more closely related to those in animals (TMEM189) than to those in Alphaproteobacteria and plants (Figure 5A). Most species have a single copy of the gene, with exceptions like zebrafish (with two copies at distinct loci; DR1 and DR2) or *A. thaliana* (with three copies; At1, At2, At3). Interestingly, within myxobacteria, CarF is largely restricted to the suborder Cystobacterineae, as are the counterparts to all of the known factors of the *M. xanthus* blue light-PPIX-^1^O_2_-CarR signaling pathway (Pérez-Castaño et al., 2022). Sequence analysis of CarF homologues and mutational analysis revealed that out of the twelve histidines in *M. xanthus* CarF, all of which would be in the cytoplasm (Figure 5B): 1) the eight that are conserved in all homologues, five of which are arranged as in motifs typically found in membrane fatty acid desaturases and hydroxylases, are essential for function (the orange-red colony color in the light, when cells have a functional CarF, provided a simple and valuable visual tool to assess function); 2) the one histidine (H113) present in homologues from metazoa, myxobacteria and *Leptospira*, but not in those from plants (where it is usually an arginine) or from Alphaproteobacteria, is also essential for function; 3) the remaining three histidines are not essential for function. One of the three homologues in *A. thaliana*, At3, turned out to be an elusive desaturase found in chloroplasts, named FAD4, which is the founding member of a novel class of plant fatty acid desaturases that generate an unusual *trans* double bond between carbons 3 and 4 from the carboxyl end of the *sn*-2 acyl chain of a specific type of phosphatidylglycerol (Gao et al., 2009; Horn et al., 2020). These observations hinted that CarF could be a special type of desaturase, as was indeed demonstrated.

Lipid analysis of wild-type versus a Δ*carF* strain, and of the latter complemented with an inducible *carF* copy in *trans*, all pointed to CarF as essential in the formation of the *M. xanthus* plasmalogen MxVEPE (Gallego-García et al., 2019). Moreover, MxVEPE was produced when purified CarF was added to cell extracts of the Δ*carF* strain together with NADPH (nicotinamide adenine dinucleotide phosphate), a required cofactor for the Δ1′-desaturase activity (Blank and Snyder, 1992). How CarF function was linked to ElbD and MXAN_1676, implicated in *M. xanthus* alkyl ether lipid and plasmalogen biosynthesis, was evaluated by comparing the response to light and the lipid profiles of the Δ*carF* strain versus strains with a single in-frame deletion of *elbD* (Δ*elbD*) or *MXAN_1676* (Δ*1676*), or with the double Δ*elbD* Δ*1676* deletion (Figure 4A). Altogether, the data indicated that whereas ElbD and to a minor degree MXAN_1676 were required for MxAEPE formation, CarF was indispensable for MxVEPE formation (Gallego-García et al., 2019). Remarkably, *M. xanthus* light-induced carotenogenesis could be chemically complemented in strains lacking MxVEPE by feeding the cells with any of three human plasmalogens tested, whose moieties at *sn*-1 and *sn*-2 (Figure 1B) differ from those in MxVEPE (Figure 3A). Furthermore, so long as CarF was present, a human alkyl ether lipid precursor could also restore the response to light, via its conversion intracellularly into the corresponding plasmalogen (Gallego-García et al., 2019). These findings firmly established that CarF is the PEDS1 desaturase, whose principal specificity determinant appears to be the alkyl ether linkage, with the type or length of the alkyl and acyl chains in the plasmanyl precursor being less crucial. They also demonstrated that plasmalogens, and not their precursors, are specifically required in the blue light-PPIX-^1^O_2_ signaling pathway leading to CarR inactivation in the *M. xanthus* carotenogenic response.

Consistent with the phylogenetic analysis of CarF homologues, those from human, mouse, zebrafish, fruit fly and worm, as well as from the animal pathogen *Leptospira*, could complement lack of CarF in *M. xanthus* (Gallego-García et al., 2019), demonstrating that these were genuine functional homologues of CarF, whereas the three plant homologues in *A. thaliana* or that from Alphaproteobacteria were not. Moreover, knocking out TMEM189 in a human cell line was shown to produce loss of plasmalogens, which were restored by supplying TMEM189 in *trans*; and mutational analysis revealed that the nine histidines equivalent to those required for CarF function were also essential for human TMEM189 to function in *M. xanthus* (Gallego-García et al., 2019). In sum, CarF PEDS1 activity was shared by its animal homologues but not by those in plants, which accords with the presence of plasmalogens in animals but not in plants, as well as with *A. thaliana* FAD4 having a different desaturase activity. Importantly, it revealed the identity of the elusive desaturase for human plasmalogen synthesis, which was substantiated soon after by two other studies demonstrating the same *via* completely different approaches (Werner et al., 2020; Wainberg et al., 2021). In one study (Wainberg et al., 2021), a statistical method to identify gene co-essentiality and predict functions of uncharacterized genes inferred TMEM189 to be PEDS1, and experimentally demonstrated it by the marked decrease in plasmalogen levels observed upon targeting *TMEM189* expression in human cell lines, as well as by the decrease in TMEM189 protein levels in a plasmalogen-deficient cell line with deficient PEDS1 activity. In the other study (Werner et al., 2020), TMEM189 emerged as the candidate by comparing gene expression data and PEDS1 activity in various human cell lines and mouse tissues, and using the criterion that PEDS1 is a probable membrane protein present only in animals (since these, but not plants or fungi, synthesize plasmalogens) with histidine motifs of the type found in lipid desaturases. This study experimentally demonstrated loss of plasmalogen and PEDS1 activity by knocking out or silencing *TMEM189* in human cell lines (and recovery by specifically expressing *TMEM189*) as well as by showing lack of plasmalogen/PEDS1 activity in mice with inactivated *TMEM189*. It further demonstrated that the same nine histidines mentioned above as crucial for CarF and human TMEM189 were also required for PEDS1 activity of the murine homologue, in which mutating H131 (the CarF H113 counterpart) strongly reduced, but did not eliminate, plasmalogen production. Besides these histidines, an aspartate (D100) and a phenylalanine (F118) conserved in CarF/TMEM189 homologues (D99 and F117, respectively, in human TMEM189) were recently shown to be required for PEDS1 activity (Werner et al., 2022).

**FIGURE 6:**
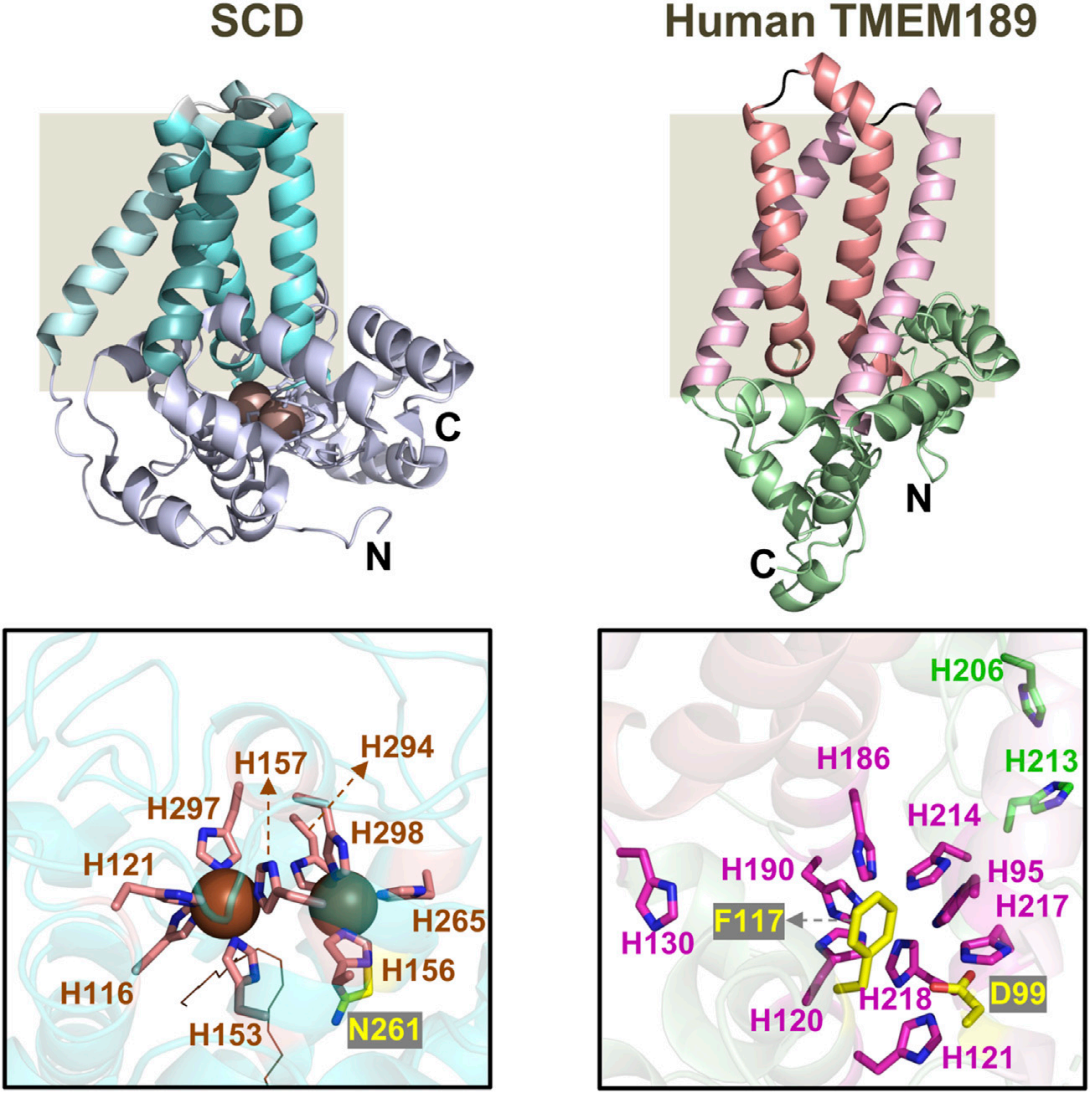
The AlphaFold2 predicted structure of human TMEM189 compared to the crystal structure of SCD. Left: Crystal structure determined for mouse stearoyl-CoA desaturase/SCD (PDB accession code; 6WF2). Right: Single-chain AlphaFold2 structure of human TMEM189 (https://alphafold.ebi.ac.uk/entry/A5PLL7). The grey box highlights the transmembrane region with four helices in each structure. Shown below are close-ups of: (left) the diiron centre (dark spheres) and the nine coordinating His and one Asn (through a water molecule) in the SCD structure (a portion of the substrate in the active site is shown by thin lines); (right) the conserved His (magenta), Asp and Phe (both yellow) essential for function, and two conserved histidines (green) not required for activity in human TMEM189 (see text).

High-resolution structures of CarF or TMEM189 can provide valuable molecular insights into how PEDS1 generates the characteristic vinyl ether bond. These studies require pure proteins but, to our knowledge, only *M. xanthus* CarF has been purified as detergent-solubilized protein, which exhibited some PEDS1 activity and appeared oligomeric with two equivalents of bound iron (Gallego-García et al., 2019). CarF thus shares the reported features of many lipid desaturases, which use a diiron center to bind and activate molecular oxygen and have an oligomeric nature proposed to be important for activity (Shanklin et al., 2009; Liu et al., 2015). Because stable expression and purification of integral membrane desaturases is generally a formidable challenge, few high-resolution structures of these proteins have been resolved. One is that of the quintessential mammalian stearoyl-CoA desaturase (SCD), which catalyzes double bond formation on saturated acyl-CoA as substrate. Interestingly, like TMEM189, the also ER-localized SCD appears to be an oligomer (Zhang et al., 2005) and its regio- and stereo-specific activity requires NADPH, cytochrome b_5_ reductase and cytochrome b_5_. The reported SCD structures are, however, monomers with four transmembrane helices and a diiron center with nine coordinating histidines and a water molecule, that is, hydrogen bonded to an asparagine residue in the cytosolic domain (Figure 6) (Bai et al., 2015; Wang et al., 2015; Shen et al., 2020). In the absence of experimentally determined structures, the single-chain AlphaFold2 structure of human TMEM189 (Figure 6) publicly available for nearly a year now (Tunyasuvunakool et al., 2021), reveals the four transmembrane helix topology experimentally demonstrated for CarF (Galbis-Martínez et al., 2008) and a spatial arrangement of the nine histidines, the aspartate and the phenylalanine required for PEDS1 activity, all of which are likely retained in the functionally equivalent CarF. This predicted fold will aid future experimental structure determinations and structure-based functional analyses of CarF/TMEM189 and their mutants.

## Lipid and Plasmalogen Signaling in *Myxococcus xanthus*

One of the clearest and most striking examples of signaling mediated specifically by plasmalogens is in the *M. xanthus* response to photooxidative stress (Gallego-García et al., 2019). It represents a new example of lipid signaling in *M. xanthus*, adding to those reported in gliding motility, chemotaxis, and the development of fruiting bodies and mature spores. Specific phosphatidylethanolamine (PE) lipids were described as chemoattractants in *M. xanthus* over 20 years ago (Kearns and Shimkets, 1998). They acted with two chemotaxis systems (Dif and Frz) that direct cell movements required for predation and fruiting body/spore formation of *M. xanthus*, a motile bacterium lacking flagella that glides slowly over solid surfaces (Kearns and Shimkets, 2001; Bonner and Shimkets, 2006; Zusman et al., 2007). Two specific PEs signaling this chemotactic response were dilauroyl (C12:0) and dioleoyl (C18:1 ω9c) PEs, which are absent in *M. xanthus* but occur in many of its prey bacteria and may be important in prey recognition. On the other hand, di [11-*Z*-hexadecenoyl] (C16:1ω5c), the third PE, is rare in bacteria but abundant in *M. xanthus* and may have roles in self-recognition (Curtis et al., 2006; Zusman et al., 2007). The two *M. xanthus* chemotaxis systems responded to these PEs via parallel, competing sensory pathways, albeit with some interdependence, to control motility (Kearns and Shimkets, 2001; Bonner and Shimkets, 2006; Zusman et al., 2007; Xu et al., 2008). Chemoreceptors present in fibrils, the extracellular appendages composed of a polysaccharide matrix and decorated with proteins of unknown function, were proposed for dilauroyl PE (Kearns and Shimkets, 2001), but those for the other two PEs are unknown. Whether PE plasmalogens also participate, and details of the molecular mechanisms by which these systems affect taxis to PE remain to be elucidated.

An essential signal (the E-signal) in the cell-cell communication/signaling required to express many genes for production of multicellular fruiting bodies and myxospores was genetically mapped to the *esg* or *bkd* loci, linking the process to branched-chain fatty acid metabolism, as mentioned earlier (Downard and Toal, 1995; Toal et al., 1995). Subsequent studies focused on the rare TG-1 alkyl ether lipid (Figure 3A), with its i15:0 *O*-alkylglycerol moiety, as important in signaling this *M. xanthus* developmental process (Bhat et al., 2014a; Ahrendt et al., 2016). Mutants in the Bkd and Aib pathway that block synthesis of the starting isovaleryl-CoA unit and, thereby, of i15:0 FA, were incapable of forming fruiting body aggregates or viable myxospores and were rescued by feeding isovalerate or i15:0 FA (Bode et al., 2009), besides TG-1 (Bhat et al., 2014a). The Elb pathway mutants, with impaired ether lipid and TG-1 formation, were delayed in fruiting body development, produced defective spores (Lorenzen et al., 2014a), and were rescued by ether lipids (Ahrendt et al., 2016). These studies thus emphasized that the i15:0 FA and the ether linkage, both present in TG-1, are important structural determinants in signaling by this alkyl ether lipid. Nonetheless, the exact identity of the ether lipid signal, whether it is TG-1 or a related lipid, has to be unequivocally established, as also the mechanisms by which this signal is detected, what its targets are, and how it is integrated into the complex cell differentiation and developmental process.

Plasmalogen-specific signaling in the *M. xanthus* photooxidative stress response is triggered by production of ^1^O_2_ (Galbis-Martínez et al., 2012; Gallego-García et al., 2019), with protein CarR as the candidate molecular target. Remarkably, both the natural *M. xanthus* plasmalogen, with *sn*-1 vinyl ether-linked and *sn*-2 ester-linked i15:0 moieties, as well as different human plasmalogens tested (bearing *sn*-1 vinyl ether-linked C18 moieties and a variety of *sn*-2 ester-linked straight chains), can signal the response (Gallego-García et al., 2019). The vinyl ether bond is therefore the crucial structural determinant required, with little or no dependence on the nature of the *sn*-1 and *sn*-2 chains. Since this photooxidative stress response leads to synthesis of carotenoids, whose role as antioxidants against ^1^O_2_ and other ROS is well-established, the part played by plasmalogens can be readily attributed to signaling rather than as antioxidant. An antioxidant role for plasmalogens, though speculative and debated (Dorninger et al., 2020; Vítová et al., 2021), is often invoked based on early findings showing the vinyl ether bond to interact with and being cleaved by ROS (Morand et al., 1988; Zoeller et al., 1988; Stadelmann-Ingrand et al., 2001).

**FIGURE 7:**
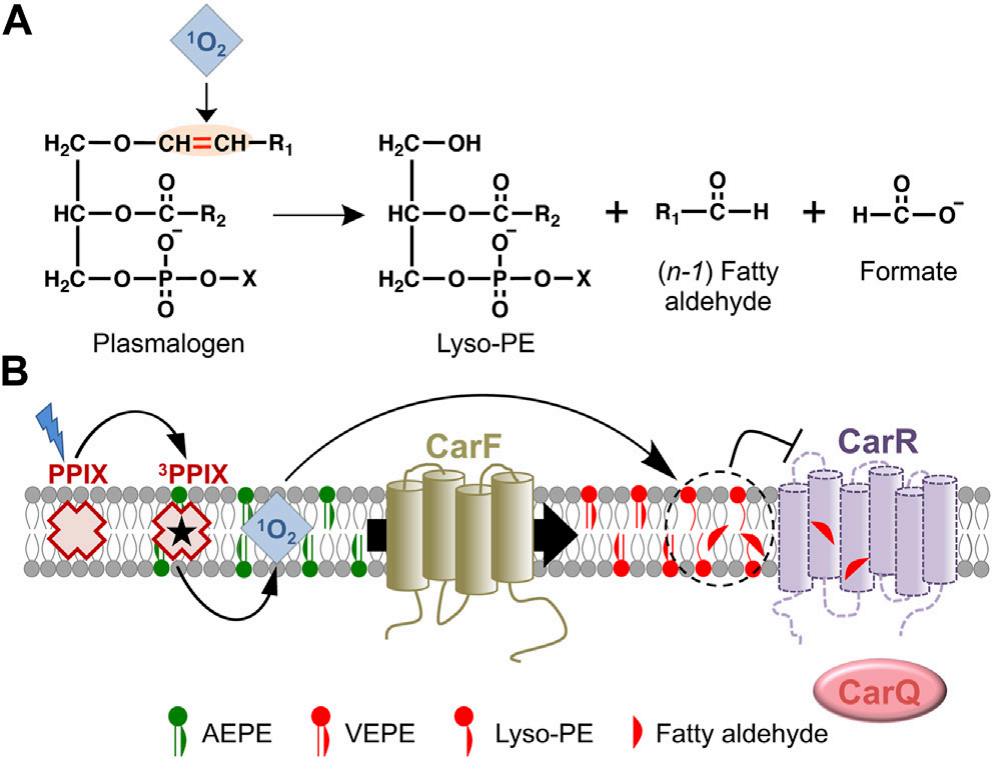
Plasmalogen signaling in the *M. xanthus* response to photooxidative stress. **(A)** Cleavage by ^1^O_2_ of the plasmalogen vinyl ether bond and its products. **(B)** Model for the plasmalogen-based signal transduction pathway leading to CarR inactivation in *M. xanthus* (arrows for activation; blunt ended for inactivation). As the PEDS1 desaturase, CarF converts (both in the dark and in the light) the plasmanyl precursor (AEPE) to plasmalogen (VEPE), which is absolutely required in the response to blue light. Generation of ^1^O_2_ upon photoexcitation of PPIX provokes cleavage of VEPE at the vinyl ether bond, generating a fatty aldehyde and lyso-PE. This may inactivate CarR directly by adduct formation with the reactive fatty aldehyde or by lipid remodeling and perturbation of the local membrane structure around CarR, leading to a conformational change and/or degradation of CarR. As a consequence, CarQ is liberated, associates with RNA polymerase (RNAP), and activates specific genes leading to carotenoid synthesis.

The molecular basis for how plasmalogens signal the presence of ^1^O_2_ in *M. xanthus* is still an open question. Reasonable conjectures can be made based on the known properties of plasmalogens, whose vinyl ether bond is susceptible of cleavage by ^1^O_2_ to yield a fatty aldehyde and a 2-acyl lyso-PE, with an *sn*-1 OH group, as illustrated in Figure 7A (Morand et al., 1988; Stadelmann-Ingrand et al., 2001). Both these cleavage products have been proposed as signaling molecules or second messengers, and their possible modes of action have been reviewed elsewhere (Jenkins et al., 2018; Dorninger et al., 2020; Ebenezer et al., 2020; Rangholia et al., 2021). Fatty aldehydes are frequent intermediates in fatty acid-fatty alcohol interconversions and are usually present at low cellular levels. They are also highly reactive and can form adducts with biomolecules such as proteins, through their cysteine, histidine or lysine residues, and these post-translational modifications may modulate cellular signaling and adaptive stress responses. Thus, an attractive hypothesis for how the *M. xanthus* photooxidative stress response may be signaled through plasmalogens is that ^1^O_2_-mediated cleavage of their vinyl ether bond produces CarR inactivation through adduct formation with the fatty aldehyde product, which may cause a CarR conformational change or trigger its degradation (Figure 7B). Nonetheless, other mechanisms cannot yet be ruled out, such as that CarR inactivation results from local perturbation of membrane structure and properties caused by plasmalogen cleavage, or more unlikely, that signaling proceeds without cleavage of the vinyl ether bond. Moreover, even though CarR is the probable direct target, the possibility of still unidentified intermediate players cannot be excluded. Future studies that exploit state-of-the-art lipidomics and click chemistry methods, combined with genetic and functional analyses, should provide the answer, and reveal the molecular intricacies underlying the *M. xanthus* plasmalogen-based signaling mechanism, designed to alert the cells that they are exposed to photooxidative stress stemming from generation of the harmful ^1^O_2_.

## Evolutionary Implications of Plasmalogen and CarF Distribution Across the Tree of Life

The unusual distribution of plasmalogens, with their presence in strictly anaerobic bacteria and in animals (vertebrates and invertebrates), and their absence in nearly all aerobic or facultatively anaerobic bacteria, fungi, or plants, led to the hypothesis that they first evolved in anaerobic bacteria, were lost in facultative and aerobic species with the advent of increasing oxygen on earth, and reappeared in animals (Goldfine, 2010). The loss in aerobes and facultative anaerobes was ascribed to adverse lipid and cell damage resulting from the increased breakdown of plasmalogens by ^1^O_2_ and other ROS with rising oxygen levels. Their reappearance in animals (with mitochondria, respiration, and molecular oxygen dependence) was attributed to the emergence of a new pathway for aerobic plasmalogen biosynthesis, distinct from the anaerobic one, and the ability to deal with cell damage from the breakdown of plasmalogens as well as to exploit their unique properties in signaling, in modulating membrane properties, and as antioxidants. Why plants lack plasmalogens was, however, unexplained, other than the aerobic pathway simply did not evolve in them. On the other hand, plasmalogen occurrence in aerobic myxobacteria was either overlooked or considered in the context of that known in anaerobic bacteria. Admittedly, at the time of the hypothesis, identifying species that made plasmalogens to gain evolutionary insights was hampered by the unknown identities of PEDS1 and of the genes for anaerobic plasmalogen biosynthesis (Goldfine, 2010). But this has now changed with the knowledge of PEDS1 identity and its remarkable conservation across a vast evolutionary distance from the aerobic myxobacteria to animals (Gallego-García et al., 2019). Moreover, the anaerobic counterpart for plasmalogen vinyl ether bond generation was also recently identified and it was found that facultative anaerobic bacteria can make plasmalogens (Jackson et al., 2021)**,** contrary to earlier thinking that this was confined to obligate anaerobes (Goldfine, 2010). These developments and the presence in plants of PEDS1 sequence homologues with a distinct and unusual desaturase activity (Gao et al., 2009) warrant a fresh look at plasmalogen biosynthesis, distribution and evolution.

In addition to the stark difference in how the signature vinyl ether bond is created in the aerobic versus the anaerobic pathways for plasmalogen biosynthesis (Figure 1C), a crucial dividing line between the two mechanisms is the use of GNP in the aerobic route (Goldfine, 2010). In the aerobic animal ether lipid/plasmalogen biosynthesis, except for vinyl ether bond formation catalyzed by the oxygen-dependent PEDS1 in a key late step, the other ER-localized reactions mirror those for ester-linked diacyl GP, and it is in the earliest steps in the peroxisome that the specialized FAR1, GNPAT and AGPS enzymes ensure formation of the defining *O*-alkyl ether linkage from GNP (Figure 2). Whether similar mechanisms operate in the aerobic myxobacterial pathway is therefore an important question. This is likely given the putative FAR1, GNPAT and AGPS domains in ElbD, MXAN_1676 (both shown to participate in alkyl ether lipid synthesis), and/or MXAN_1,675 (Figure 3B), although these and other steps and enzymes need to be fully characterized (Lorenzen et al., 2014a; Gallego-García et al., 2019). Strikingly, CarF homologues are found in just one myxobacterial suborder, the Cystobacterineae (Pérez-Castaño et al., 2022), but *elbD* and *elbE* (forming a cluster with *elbB* and *elbC*, or unlinked to them) are found in all three suborders (Lorenzen et al., 2014a). Consequently, alkyl ether lipids may be common across myxobacteria, but plasmalogens are most likely restricted to a single suborder. Interestingly, beyond myxobacteria, true CarF homologues have been reported only in *Leptospira* (Gallego-García et al., 2019), and the genome of *L. interrogans*, a Gram-negative obligately aerobic animal pathogen, reveals an annotated AGPS but no FAR1. Whether it is capable of *ab initio* plasmalogen biosynthesis or requires the animal host for suitable precursors or plasmalogens is unknown. On the other hand, some bacteria from other phyla that lack CarF and are anaerobes, animal pathogens, or extremophiles possess ElbD-like homologues, but almost all of these lack a FAR1 domain (Lorenzen et al., 2014a), and little or no lipid analysis is available for these bacteria.

Generally, the chemistry and composition of eukaryotic membrane lipids are far more like those in bacteria than in archaea (Lombard et al., 2012; López-García and Moreira, 2020; Hoshino and Gaucher, 2021). Myxobacteria, in particular, stand out as a singular group among prokaryotes, with several typically “eukaryotic” lipids that include phosphatidylinositol, sphingolipids and steroids (or their molecular surrogates called hopanoids) besides, of course, ether lipids/plasmalogens (Ring et al., 2009; Lorenzen et al., 2014a; Lorenzen et al., 2014b; Sohlenkamp and Geiger, 2016; Gallego-García et al., 2019; Hoshino and Gaucher, 2021). Notably, counterparts to both the eukaryotic oxygen-dependent steroid and its isoprenoid precursor mevalonate biosynthesis pathways are near exclusive to aerobic myxobacteria (Hoshino and Gaucher, 2021; Pérez-Castaño et al., 2022). Thus, rather than an anomaly, animal-like aerobic plasmalogen biosynthesis in myxobacteria constitutes yet another candidate to add to a growing list of eukaryotic-like factors and traits. This list has led to the hypothesis that symbiosis or syntrophy of an ancestral host myxobacterium, an Asgard-like archaeon as future nucleus, and an alpha-proteobacterium as future mitochondrion may have been a crucial early event in the origin of eukaryotes (López-García and Moreira, 2020; Hoshino and Gaucher, 2021). If so, it would point to a myxobacterial source in the beginnings of aerobic plasmalogen biosynthesis. Studies to further elucidate the pathway in myxobacteria may therefore reinforce such conjectures on its origins and provide evolutionary insights into extant mechanisms.

## Concluding Remarks

Unmasking the identity of the long-elusive oxygen-dependent PEDS1 and its largely unanticipated occurrence in bacteria is arguably a turning point and a thrust to studies on the biosynthesis, functions, origins, evolution, and roles in disease of the enigmatic plasmalogens. It may also provide a valuable synthetic biological tool for aerobic plasmalogen synthesis. Strikingly, discovery of myxobacterial PEDS1 simultaneously uncovered a novel and specific role of plasmalogens as exquisitely sensitive signaling molecules acting with ^1^O_2_ in a photooxidative stress response. Deciphering the complete pathway(s) in the biosynthesis of plasmalogens, how they signal, and what their target molecules and processes are in myxobacteria may reveal novel molecular mechanisms. These may be universal, perhaps extensible, to other cell types, notably mammalian, and shed light on conserved as well as evolutionary aspects of plasmalogens in prokaryotes and eukaryotes.
